# Inhibition of protein tyrosine phosphatase improves mitochondrial bioenergetics and dynamics, reduces oxidative stress, and enhances adipogenic differentiation potential in metabolically impaired progenitor stem cells

**DOI:** 10.1186/s12964-021-00772-5

**Published:** 2021-11-03

**Authors:** Katarzyna Kornicka-Garbowska, Lynda Bourebaba, Michael Röcken, Krzysztof Marycz

**Affiliations:** 1 Department of Experimental Biology, Wroclaw University of Environmental and Life Sciences, Norwida 27B Street, A7 building, 50-375 Wroclaw, Poland; 2 International Institute of Translational Medicine, Malin, Jesionowa 11, 55-114 Wisznia Mała, Poland; 3 Faculty of Veterinary Medicine, Equine Clinic-Equine Surgery, Justus-Liebig University, 35392 Giessen, Germany

**Keywords:** Progenitor stem cells, Adipogenesis, PTP1B, LMPTP, Mitochondria

## Abstract

**Background:**

Protein tyrosine phosphatase 1B (PTP1B) and low molecular weight protein tyrosine phosphatase (LMPTP) are implicated in the development of metabolic disorders. Yet, their role in progenitor stem cell adipogenic differentiation and modulation of mitochondrial dynamics remains elusive.

**Methods:**

In this study, we decided to investigate whether inhibition of PTP1B and LMPTP enhance adipogenic differentiation of metabolically impaired progenitor stem cells via modulation of mitochondrial bioenergetics and dynamics. Cells were cultured under adipogenic conditions in the presence of PTP1B and LMPTP inhibitors, and were subjected to the analysis of the main adipogenic-related and mitochondrial-related genes using RT-qPCR. Protein levels were established with western blot while mitochondrial morphology with MicroP software.

**Results:**

Selective inhibitors of both PTP1B and MPTP enhanced adipogenic differentiation of metabolically impaired progenitor stem cells. We have observed enhanced expression of PPARy and adiponectin in treated cells. What is more, increased antioxidative defence and alternations in mitochondrial bioenergetics were observed. We have found that inhibition of PTP1B as well as C23 activates oxidative phosphorylation and enhances mitochondrial fusion contributing to enhanced adipogenesis.

**Conclusions:**

The presented data provides evidence that the application of PTP1B and LMPTP inhibitors enhances adipogenesis through the modulation of mitochondrial dynamics.

**Video abstract**

**Supplementary Information:**

The online version contains supplementary material available at 10.1186/s12964-021-00772-5.

## Background

Prevalence of metabolic syndrome has rapidly increased among humans and domestic animals, becoming the noninfectious epidemic of the XXI century. In horses, the set of distinct metabolic alternations is entitled as equine metabolic syndrome (EMS), and similar to human Syndrome X (MetS) is recognized as a cluster of associated conditions including insulin resistance/insulin dysregulation, systemic inflammation, hyperlipidemia or cardiovascular complications (*laminitis* in horses) [1, 2]. EMS develops due to sedentary life style, lack of physical activity, and finally excess energy consumption with low fiber amount and energy flux disruptions. It is estimated that more than 40% of US adults are physically inactive, which is associated with so-called western lifestyle [3]. According to the National Center for Health Statistics, Division of Health Interview Statistics, one-third of adults in the US suffer from MetS and 30% of the UK population of horses are diagnosed as insulin resistant [4, 5]. This alarming data regarding disorders affecting both human and equine patients, highlighting the urgent need to develop new pharmaceutical interventions for MetS and EMS treatment.

In the light of the recent findings, adipose tissue is recognized as a critical endocrine component of both MetS and EMS development, although in horses obesity is not a crucial factor for the diagnosis of metabolic syndrome [6, 7]. Adipose tissue in responding to chronic excessive energy supply increases its volume (hypertrophy) or increases the size of single adipocytes (hyperplasia) [8]. In these processes, progenitor stem cells (ASCs), which reside within it, play an important regulatory role since they give rise to new populations of adipocytes and thus are directly involved in adipose tissue metabolism [6]. Available data indicate that ASCs become an "sensitive" pool of progenitor cells, which under unfavorable conditions might negatively affect the adipose tissue cellular compartment by secretion of multiplate factors including proinflammatory cytokines or hormones [9, 10]. Our and other research have shown that ASCs isolated from EMS horses are losing their pro-regenerative phenotype and as a consequence might lead to the development of dysfunctional adipose tissue [11, 12]. Recently, it was suggested that ASCs from EMS horses are characterized by impaired immunomodulatory action, which deprives adipose tissue of natural anti-inflammatory protection [2, 13]. Due to their physiological role, ASCs are directly involved in adipose tissue remodeling due to abundant production of hormones, proinflammatory cytokines, metabolites, and finally reduced/impaired adipogenic differentiation potential leading to the development of dysfunctional adipose tissue [14].

Mitochondria, which are recognized as a “powerhouse unit” of the cell, converting chemical energy into ATP, stands as a key factor during adipogenesis [15]. Fatty acid β-oxidation, tricarboxylic acid (TCA) cycle, pyruvate oxidation, and oxidative phosphorylation (OXPHOS) which occur in mitochondria, affect single cell performance and metabolic conditions. Moreover, undifferentiated ASCs undergo several metabolic changes mediated by mitochondrial activity, which includes the activity of cAMP-responsive element-binding protein (CREB), CCAAT/enhancer-binding protein (C/EBP) family members and peroxisome proliferator-activated receptor γ (PPARγ)-master regulators of adipogenesis [16, 17]. The dysregulation of these genes expression directly affects mitochondria biogenesis and dynamics mediated by fusion and fission processes [18]. Recently, we have demonstrated that mitochondrial metabolism is deteriorated in ASC isolated from type two diabetes conditions in human patients and EMS horses [19–21]. Increased production of reactive oxygen species (ROS) or nitric oxide (NO) together with decreased antioxidative properties impairs the multilineage differentiation potential of ASCs, leading to impaired differentiation potential [22]. The deteriorated ASCs metabolism caused by mitochondrial defects might be recognized as an important component of insulin resistance development in ASC cells. Moreover, adipose tissue hyperplasia and inflammation mainly results from mitochondrial dysfunction [23]. Mitochondrial biogenesis and dynamics are enhanced during ASCs differentiation, modulating metabolic homeostasis in adipose tissue [24, 25]. Our earlier findings demonstrated that deterioration of mitophagy, the process allowed to eliminate defective mitochondria, occurs during EMS and diabetes, driving the progression of insulin resistance [12, 20].

Insulin receptor substrate pathway (IRSP) is directly involved in the control of anabolic responses in adipose tissue by stimulating glucose and free fatty acid uptake, de novo synthesis of fatty acids and lipolysis [26]. Insulin mediates and regulates ASCs growth as well as differentiation potential by the activation of several transcription factors including SREBP-1c and PPARγ. Thus, precise modulation of IRSP is fundamental to ensure proper insulin response. The IRSP pathway has been a major focus of research in rodents and humans, although no studies have been performed in horses suffering from EMS. Any alterations that disturb the pathway homeostasis may lead to insulin resistance [27]. Negative regulation of insulin action includes inhibitory mechanisms based on dephosphorylation of proteins involved in signal transmission. Protein tyrosine phosphatases (PTPs) are known to inactivate IRP via its dephosphorylation and thus contributing to insulin resistance [28]. Numerous studies focus on protein tyrosine phosphatase 1B (PTP1B), a well-known target for diabetes and obesity management. It was demonstrated that PTP1B knockout mice displayed enhanced insulin sensitivity and IRP phosphorylation, being resistant to high-fat diet (HFD)-induced obesity [29]. This corelates with the study performed by Stull et al. [30], who revealed that PTP1B gene expression and protein abundance is increased in the biopsied skeletal muscles in humans with type two diabetes (T2D). In recent years, a lot of pharmaceutical companies have developed various PTP1B inhibitors as drug candidates for therapy of T2D in clinical trials, including ertiprotafib, ISIS 113715 and MSI1436-trodusquemine. Interestingly, recently Stanford et al. [31] indicated the role of low molecular weight protein tyrosine phosphate (LMPTP) in the development of diabetes and synthetized a novel, specific LMPTP inhibitor “compound 23-C23” (N,N-diethyl-4-(4-((3-(piperidin-1-yl)propyl)amino)quinolin-2-yl) benzamide). C23 is a small molecule inhibitor with a novel uncompetitive mechanism, a unique binding site at the opening of the catalytic pocket, and an exquisite selectivity over other phosphatases. Application of the LMPTP inhibitor in DIO mice increased liver insulin receptor (IRP) phosphorylation and reversed HFD-induced diabetes [31] which highlights its therapeutic potential against insulin resistance and diabetes.

In this study, we aim to verify whether in vitro application of MSI1436 and C23 improves mitochondrial biogenesis and dynamics, reduces oxidative stress, and improves adipogenic differentiation potential in progenitor stem cells isolated from equine metabolic syndrome individuals. We have demonstrated that both chemicals reduce oxidative stress, improve antioxidative defense, and finally, through modulation of mitochondrial biogenesis and dynamics, improve adipogenic differentiation in ASCs from EMS horses.

## Methods

All chemicals and reagents were obtained from Sigma Aldrich (Poznań, Poland), unless otherwise stated. Cell culture reagents were purchased from BioWest (VWR International, Gdańsk, Poland).

### Isolation and culture of equine adipose tissue derived mesenchymal stem cells

Equine ASCs cells were isolated from subcutaneous adipose tissue collected from the tail base area of adult healthy (HE) and EMS horses, under local anesthesia induced by 2% lidocaine (Polfa S.A., Warsaw, Poland) as previously described [32]. Detailed characterization of animals has been described in our previous studies [33]. Qualification to the experimental groups was performed based on (i) extensive interviews with owners, (ii) measurement of body weight, (iii) estimation of body condition score (BCS) and cresty neck scoring system (CNS), (iv) palpation and visual assessment of the hoof capsule, (v) X-ray examination, (vi) resting insulin levels, (vii) combined glucose-insulin test (CGIT), and (viii) LEP concentration Harvested fat samples were profusely washed using 1% antibiotics Hanks’ Balanced Salt Solution (HBSS) mixture to avoid any microbial contamination. Adipose tissue sections were afterwards finely minced using a sterile scalpel blade, then underwent enzymatic digestion using 0.1% collagenase type I (0.1 mg/mL) for 40 min at 37 °C and 5% CO_2_, and finally centrifuged at 1200 × g for 10 min. The obtained cell pellet was resuspended in Dulbecco’s modified Eagle’s medium (DMEM) containing 1000 mg/L glucose supplemented with 5% of foetal bovine serum (FBS), and 1% of penicillin and streptomycin (PS) solution in culture flasks. Isolated cells were cultured in a humidified CO_2_ incubator (37 °C, 5% CO_2_, 95% air atmosphere), passaged every three days (80 – 90% of confluence) using an Accutase® solution and used for experimental purposes starting from the third passage.

Cellular population purity as well as ASCs cells phenotyping were evaluated using a Fluorescent-Activated cell sorting technique (BD FACSCalibur, Becton Dickinson, Franklin Lakes, New Jersey, USA) and flow cytometry analysis with fluorochrome conjugated monoclonal antibodies (anti-CD105, Acris, Herford, Germany, SM1177PT; anti-CD45, Novus Biologicals, Littleton, Colorado, USA, NB1006590APC, anti-CD44, R&D Systems, Minneapolis, Minnesota, USA, MAB5449, anti-CD90, ab225; Abcam, Cambridge, UK) respectively. Multipotency character of isolated ASCs was confirmed after osteogenic, chondrogenic, and adipogenic differentiation of cells cultured in StemXVivo kits (R&D System). All above-mentioned techniques and related results were extensively described and shown in previous publications [33–36].

### Adipogenic differentiation of equine ASCs cells

To achieve adipogenic differentiation, ASCs cells derived from either healthy (HE) or EMS horses were firstly seeded onto culture plates in regular DMEM culture medium until the cells became confluent. To induce adipocyte differentiation, post-confluent ASCs cells were stimulated using the StemPro™ Adipogenesis Differentiation Kit (Gibco™, Thermo Fisher Scientific, Poland) containing 0.5 mM of 1-methyl-3-isobutylxantine (IMBX), 0.5 mM of dexamethasone, 6.25 ug/ml of insulin, 60 uM of indomethacin, 50 ug/ml of gentamicin, 10% of FBS, and DMEM with Nutrient F-12 Ham, according to manufacturer’s instructions. Cultivation under adipogenic inductive conditions was carried out for 14 days in the presence or absence of MSI-1436 and/or Compound 23 inhibitors at a concentration of 1 µM, with the replacement of culture media every three days.

In the meantime, undifferentiated HE and EMS cells were maintained in the basal culture medium consisting of low glucose DMEM and 5% FBS and 1% P/S, and were used as negative controls for all experiments.

At the 15^th^ day of differentiation process, all cultures were stopped and subjected to further analysis described below.

### Changes in mitochondrial membrane potential (MMP) detection assay

Differentiated and undifferentiated ASCs cells were analyzed for their mitochondrial membrane potential (MMP) using the Muse™ MitoPotential Assay kit (Cat. No. MCH100110, Merck Millipore, Darmstadt, Germany). Briefly, EMS and healthy differentiated or undifferentiated cells were firstly washed with HBSS, resuspended in 1X assay buffer, and labelled with the provided fluorescent dyes for 30 min in a 37 °C and 5% CO_2_ incubator. The percentage of total depolarized cells (depolarized live + depolarized dead) was afterwards evaluated by the mean of a Muse Cell Analyzer (Merck Millipore, Darmstadt, Germany).

### MitoTracker Red staining for confocal imaging

Mitochondrial network was visualized using confocal microscopy (Observer Z1 Confocal Spinning Disc V.2, Zeiss with live imaging chamber). Living mitochondria were stained using the MitoRed fluorescent dye (1:1000 in medium) at the 15th day of differentiation, for 30 min at 37 °C in a CO_2_ incubator. Excess dye was then removed by washing the samples with HBSS, and the cells were subsequently fixed with 4% PFA during 40 min at room temperature. Nuclei were labelled with diamidino-2-phenylindole (DAPI), using the ProLong™ Diamond Antifade Mountant with DAPI (Invitrogen™, Poland). Images were subjected to MircoP software (http://bmi.ym.edu.tw/jypeng/).

### Fluorescent detection of intracellular Lipid Droplets

Neutral lipid droplets in all cultured ASCs cells were analysed using the fluorescent HCS LipidTOX™ Green Neutral Lipid Stain (Invitrogen Life Technologies, Warsaw, Poland) for cellular imaging. Staining was performed following the protocol of the manufacturer as described elsewhere [37]. ASCs cells were fixed in 4% paraformaldehyde for 40 min prior to staining with LipidTOX™ Green during 20 min at room temperature. After three HBSS washes, the nuclei were counterstained with DAPI using the ProLong™ Diamond Antifade Mountant with DAPI (Invitrogen Life Technologies, Warsaw, Poland). Photomicrographs were captured using a confocal microscope (Observer Z1 Confocal Spinning Disc V.2, Zeiss with a live imaging chamber). The obtained photomicrographs were then merged and analysed using the ImageJ software (Bethesda, MD, USA).

### Crude Mitochondria isolation for transcriptomic analysis

To investigate the crosstalk between PTP inhibition and mitochondrial dynamics changes during the adipogenesis process, total mitochondria were isolated from the studied equine ASCs cells using the commercial Thermo Scientific™ Mitochondria Isolation Kit for Cultured Cells (Thermo Fisher Scientific, Warsaw, Poland), according to the manufacturer’s instructions. Briefly, all differentiated, treated, untreated and undifferentiated cells were collected from culture flasks, washed three times with cold HBSS, and pelleted by centrifugation at 850×*g* for 2 min. After discarding the supernatants, cells were sequentially lysed using the provided lysis reagents containing a protease inhibitor cocktail (1:1000) on ice. Remaining cellular, derbies, and cytosolic fractions were discarded by centrifuging cell lysates at 700×*g* for 10 min at 4 °C. Obtained total mitochondria-rich supernatants were subsequently centrifuged at 12,000×*g* for 15 min at 4 °C. The final mitochondria pellets were then resuspended in TRIzol reagent for RNA isolation.

### RNA preparation and quantitative RT-PCR for gene expression analysis

Total RNA of all groups of cells was isolated using the TRIzol method according to the manufacturer’s instructions. RNA purity and concentration were assayed by means of a nanospectrophotometer (WPA, Biowave II, Germany). Genomic DNA (gDNA) digestion and cDNA synthesis were performed using a PrimeScript™ RT Reagent Kit with a gDNA Eraser (TaKaRa, Gdańsk, Poland) and using a T100 Thermal Cycler (Bio-Rad, Hercules, CA, USA) in accordance to the manufacturer’s recommendations.

Prior to quantitative RT-PCR analysis, mt-cDNA samples were preamplified using a pool of targeted mitochondrial-related gene primers under the following cycling conditions: 95 °C for 2 min, followed by 18 cycles at 95 °C for 3 s, annealing for 3 min and 72 °C for 3 s.

Expression levels of targeted genes (Table 1) were investigated through real-time reverse transcription polymerase chain reaction (RT-PCR), using a SensiFAST SYBR Green Kit (Bioline, London, UK) in a CFX Connect™ Real-Time PCR Detection System (Bio-Rad). Reactions were performed in a 10 µl volume containing 150 ng cDNA, and were amplified under the following cycling conditions: 95 °C for 2 min, followed by 40 cycles at 95 °C for 15 s, annealing for 15 s, and elongation at 72 °C for 15 s. All results were normalized to glyceraldehyde 3-phosphate dehydrogenase (GAPDH) expression. The relative expression level was calculated using the 2^−ΔΔCQ^ method [38].

**Table 1:** Sequence of primers used in qPCR

Gene	Primer	Sequence 5′–3′	Amplicon length (bp)	Accession No
*PPARγ*	F: R:	TCCCTGTTTGTGTACAGCCTT CTCCATGGCTGATTTCCCCT	191	XM_014846252.1
*ADIPOQ*	F: R:	GGAGATCCAGGTCTTGTTGG TCGGGTCTCCAATCCTACAC	162	XM_014843352.1
*Lep*	F: R:	CACACGCAGTCAGTCTCCTC CGGAGGTTCTCCAGGTCAT	176	XM_014854289.1
*SREBP-1c*	F: R:	TCAGCGAGGCGGCTTTGGAGCAG CATGTCTTCGATGTCGGTCAG	80	XM_008542859.1
*SOD1 (Cu/Zn SOD)*	F: R:	CATTCCATCATTGGCCGCAC GAGCGATCCCAATCACACCA	130	NW_001867397.1
*SOD2 (Mn SOD)*	F: R:	GGACAAACCTGAGCCCCAAT TTGGACACCAGCCGATACAG	125	NW_001867408.1
*CAT*	F: R:	ACCAAGGTTTGGCCTCACAA TTGGGTCAAAGGCCAACTGT	112	XM_014851065.1
*PPARGC1β*	F: R:	CCTCAACTATCTTGCCGACACC ATGGGTTCAGTCTCGGGGT	165	XM_023617445.1
*OXA1L*	F: R:	GACCTAGAAACCGTGGGACG GGAAGATCACTTGGCTCCCC	105	XM_008528958.1
*MRPL24*	F: R:	ATGATCCCTAGCGAAGCACC TGTAGAGACTCGTACCCGCT	123	XM_001500466.4
*MTERF4*	F: R:	CGCCACCTCCGTGCTATG CCCAAATGAGGGGCATCAGG	147	XM_023644068.1
*PUSL1*	F: R:	TCAGCCACTTCCAGGACCTA AGCCACATCCAAGCTGTCTG	120	XM_023636046.1
*TFAM*	F: R:	ATGATGGCTTTGAGTCCAGG CTAGATGATGGCGGGAGACTT	154	XM_023643450.1
*NDUFA9*	F: R:	TTGGTATTCAGGCCACACCC GCTGGCTTCACGTCTTCAAC	103	XM_001494601.4
*UQCRC2*	F: R:	TGCTTCGTCTTGCATCCAGT AACTCCGGTGACGTGGTAAC	193	XM_001494381.5
*COX4I1*	F: R:	GAATAGGGGCACGAACGAGT GCCACCCACTCCTCTTCAAA	138	XM_023637444.1
*COX4I2*	F: R:	CCCCACCCCCAGATGTTCT CGTGGTAGTTGGTGTAGGGG	136	XM_005604417.3
*PIGBOS1*	F: R:	GTTGGGGTGGCTCAGATCAA ACCCCTCCTTTACCGCTACT	126	XM_014733689.2
*MIEF1*	F: R:	GTGAGCGCAAAGGCAAGAAA CTTAACCGCCAGTGTAGCGA	128	XM_023631522.1
*PINK1*	F: R:	GCACAATGAGCCAGGAGCTA GGGGTATTCACGCGAAGGTA	289	XM 014737247.1
*PARKIN*	F: R:	TCCCAGTGGAGGTCGATTCT CCCTCCAGGTGTGTTCGTTT	218	XM 014858374.1
*MFN1*	F: R:	AAGTGGCATTTTTCGGCAGG TCCATATGAAGGGCATGGGC	217	XM 001495170.5
*MFN2*	F: R:	AGGTGAAGTCAGAATTGGTGGA CTTCACAGGGGTGGCATCAT	129	XM_023635773.1
*Fis*	F: R:	GGTGCGAAGCAAGTACAACG GTTGCCCACAGCCAGATAGA	118	XM 001504462.4
*MIRO1*	F: R:	GATCCTGCTGGTGGGAGAAC GGGAGGAACCTCTTCTGGGA	88	XM_023651639.1
*OPA1*	F: R:	CTTCTCTTGTTAGGTTCACCTGG TGTAAGAGAATGAGCTCACCAAG	110	XM_003363363.4
*DRP1*	F: R:	CAGCTAGTCCACGTTTCACC CCCTTTAGAAAGGTGTCTTGAGT	96	XM_023643346.1
*LC3*	F: R:	TTCTGAGACACAGTCGGAGC CTTTGTTCGAAGGTGTGGCG	128	XM_001493613.6
*Beclin*	F: R:	GATGCGTTATGCCCAGATGC AACGGCAGCTCCTCTGAAAT	233	XM_014833759.1
*Lamp2*	F: R:	GCACCCCTGGGAAGTTCTTA ATCCAGCGAACACTCTTGGG	147	XM_014729146.2
*GAPDH*	F: R:	GATGCCCCAATGTTTGTGA AAGCAGGGATGATGTTCTGG	250	NM_001163856.1

Sequence: amplicon length and access numbers of the primer sets. *PPARγ*: Peroxisome proliferator-activated receptor gamma; *ADIPOQ*: Adiponectin; *Lep*: Leptin; *CEBPA*: CCAAT/enhancer-binding protein alpha; *SREBP-1C*: Sterol Regulatory Element Binding Protein-1c; *AKT1*: Serine-threonine protein kinase 1; *AKT2*: Serine-threonine protein kinase 2; *INSR*: Insulin receptor; *IRS1*: Insulin Receptor substrat 1; *PI3K*: Phosphoinositide 3-Kinase*; SHBG*: Sex hormone binding globulin; *AHSG*: Alpha 2-HS Glycoprotein; *P53*: tumor suppressor p53; *Bcl-2*: B-cell lymphoma 2; *Bax*: BCl-2 associated X protein; *p21*: Cyclin-dependent kinase inhibitor 1; *Casp3*: Caspase 3; *Casp9*: Caspase 9; *Perk*: PRKR-like endoplasmic reticulum kinase; *Chop*: C/EBP homologous protein; *Atf6*: Activating transcription factor 6; *Ire1*: Inositol-requiring enzyme; *Xbp1*: X-box binding protein 1*Sod1 (Cu/Zn SOD):* Copper-zinc-dependant superoxide dismutase *(CuZnSOD*; *Sod2 (Mn SOD):* Manganese-dependent superoxide dismutase (MnSOD*); CAT: Catalase; PPARGC1β:* Peroxisome proliferator-activated receptor γ coactivator 1-beta; *OXA1L:* OXA1L, mitochondrial inner membrane protein; *MRPL24*: Mitochondrial Ribosomal Protein L24; *MTERF4*: Mitochondrial Transcription Termination Factor 4; *PUSL1*: Pseudouridine Synthase Like 1; *TFAM*: Mitochondrial transcription factor A; *NDUFA9*: NADH ubiquinone oxidoreductase subunit A9; *UQCRC2*: Ubiquinol Cytochrome C Reductase Core Protein 2; *COX4I1*: Cytochrome c oxidase subunit 4 isoform 1; *COX4I2*: Cytochrome c oxidase subunit 4 isoform 2; *PIGBOS1*: PIGB, opposite strand protein 1; *MIEF1*: Mitochondrial Elongation Factor 1; *PINK1*: PTEN-induced kinase 1; *PARKIN*: Parkin RBR E3 ubiquitin protein ligase (PARK2); *MFN1*: Mitofusin 1; *MFN2*: Mitofusin 2; *FIS1*: mitochondrial fission 1 molecule; *MIRO1*: Ras Homolog Family Member T1; *OPA1*: OPA1, Mitochondrial Dynamin Like GTPase; *DRP1*: Dynamin-1-like protein; *GADPH*: glyceraldehyde-3-phosphate dehydrogenase

### Mitochondrial protein profiling

Relative expression of mitochondrial dynamics-related proteins was investigated by western blotting. Briefly, all groups of cells were detached from culture dishes at the 15th day of differentiation induction, and lysed in RIPA buffer (50 mmol/L Tris pH 7.4, 150 mmol/L NaCl, 0.1% SDS, 0.5% sodium deoxycholate, 1% Triton X-100, protease cocktail, 1 mmol/L PMSF, 10 mmol/L sodium azide, 10 mmol/L sodium ascorbate, and 5 mmol/L Trolox) containing protease inhibitor cocktail on ice. After centrifugation at 20 min, 12,000 × g, 4 °C to remove insoluble materials, the supernatants were collected to fresh tubes and stored at − 80 °C until further use. The protein concentration was established using the Pierce™ Bicinchoninic Acid (BCA) Protein Assay Kit (Life Technologies, USA). For protein separation, cell lysates were firstly diluted with 4 × Laemmli loading buffer (Bio-Rad, USA) and denatured for 5 min at 95 °C. Samples were then subjected to SDS–polyacrylamide gel electrophoresis at 100 V for 90 min in Tris/glycine/SDS buffer using Mini-PROTEAN Tetra Vertical Electrophoresis Cell (Bio-Rad, USA) and transferred onto polyvinylidene difluoride (PVDF) membranes (Bio-Rad, USA) using a transfer apparatus Mini Trans-Blot® Cell (Bio-Rad, USA) at 100 V, 250 mA for 45 min at 4 °C in Tris/glycine buffer/methanol. The membranes were subsequently blocked using a 5% nonfat milk solution prepared in TBST. Each protein was detected by overnight incubation at 4 °C with primary antibodies listed in Table 2 and HRP-conjugated secondary antibodies (dilution 1:2500 in TBST, 1 h incubation at room temperature). Chemiluminescent signals were detected using ChemiDoc MP Imaging System (Bio-Rad, USA) and quantified with Image Lab Software (Bio-Rad, USA).

**Table 2:** List of antibodies employed for protein profiling using western blot analysis

Antibody	Dilution	Catalog no
*Pink1*	1:1000	Biorbyt, orb331223
*Mfn1*	1:1000	Biorbyt, orb11040
*Mff*	1:1000	Biorbyt, orb325479
*GAPDH*	1:2000	Biorbyt, orb323147

*Pink1:* PTEN-induced kinase 1; *Mfn1*: Mitofusin-1; *Mff*: Mitochondrial fission factor; *GADPH*: glyceraldehyde-3-phosphate dehydrogenase

### Statistics

All statistical analyses and graphical representations were performed using GraphPad Prism (San Diego, CA, USA). Statistical differences were established with a one-way analysis of variance (ANOVA) followed by Bonferroni post hoc multiple comparison test, as indicated. All p values lower than 0.05 (*p* < 0.05) are summarized with one asterisk or number sign (*, #), those at *p* < 0.01 use two asterisks or number signs (**, ##), and those at *p* < 0.001 have three asterisks or number signs (***, ###).

## Results

### Analysis of mitochondria metabolism in undifferentiated ASCs

**Fig. 1:**
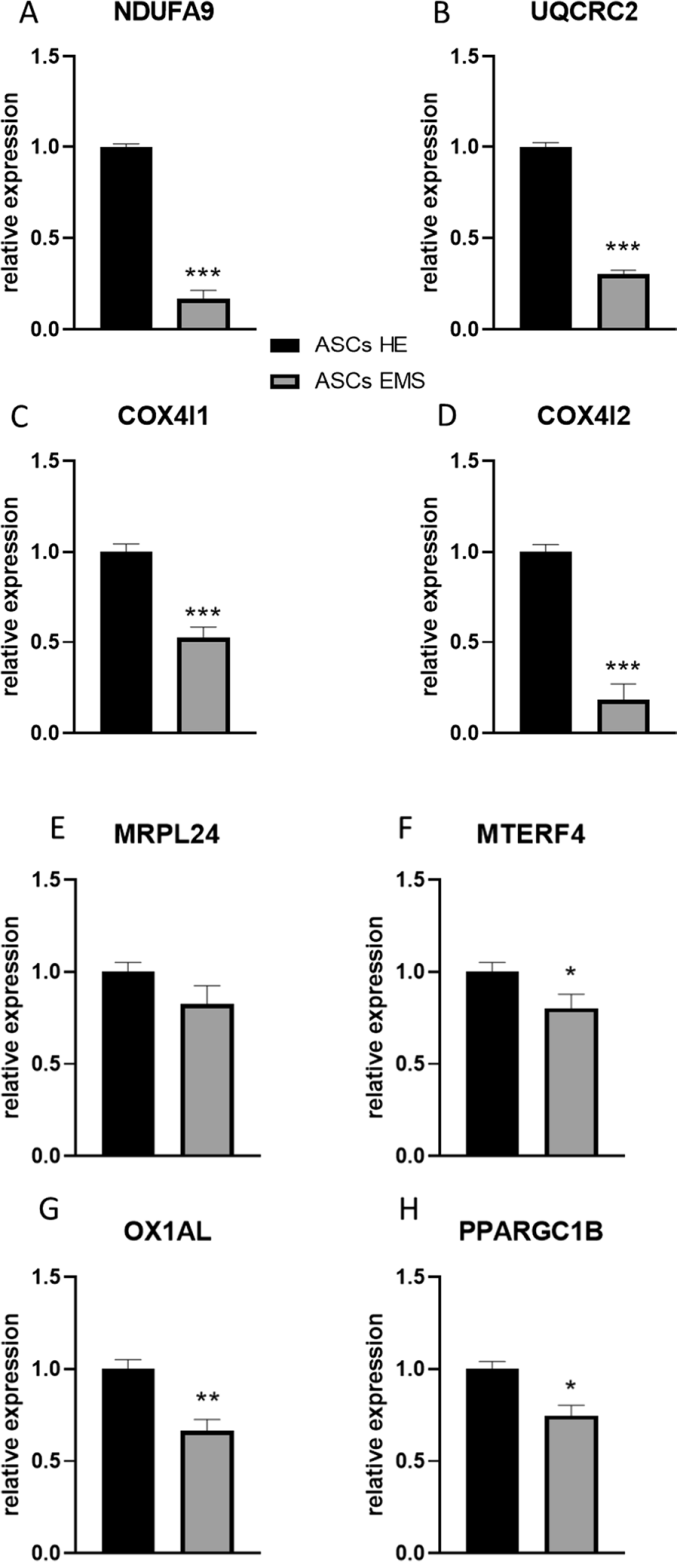

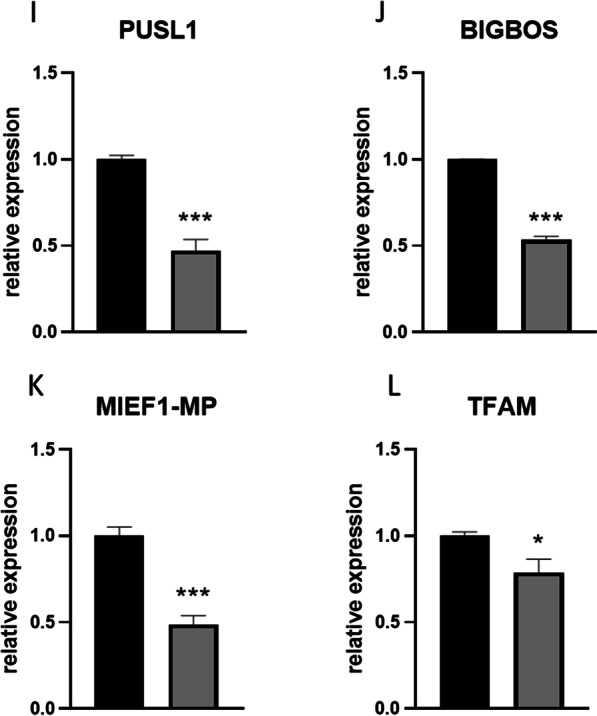
Analysis of mitochondria metabolism in undifferentiated ASCs. Expression of NDUFA9 (**a**), UQCRC2 (**b**), COX4I1 (**c**), COX4I2 (**d**), MRPL24 (**e**), MTERF4 (**f**), OX1AL (**g**), PPARGC1B (**h**), PUSL1 (**i**), BIGBOS (**j**), MIEF1-MP (**k**) and TFAM (**l**) was investigated with RT-qPCR. Results are expressed as mean ± SD; *n* = 3.∗*p* < 0.05, ∗∗*p* < 0.01, and ∗∗∗*p* < 0.001

Using RT-qPCR, we analysed the expression of genes related to mitoribosomes and the mitochondrial oxidative phosphorylation (OXPHOS) system in undifferentiated cells. NDUFA9 (Fig. 1a), UQCRC2 (Fig. 1b), COX4I1 (Fig. 1c), and COX4I2 (Fig. 1d) expression was significantly (*p* < 0.001) downregulated in ASCs EMS. On the other hand, no significant differences were noted in the expression of MRPL24 (Fig. 1e). mRNA levels of MTERF4 (Fig. 1f) and OX1AL (Fig. 1g) were significantly (*p* < 0.05 and *p* < 0.01 respectively) diminished in ASCs EMS when compared to ASCs HE. Expression of PPARGC1B (Fig. 1h) was also downregulated in that group (*p* < 0.05). ASCs EMS were characterised by downregulation of PUSL1 (Fig. 1i), BIGBOS (Fig. 1j), MIEF1-MP (Fig. 1k) (*p* < 0.001) and TMFAM (Fig. 1l) (*p* < 0.05) expression as well.

### Analysis of mitochondrial dynamics in undifferentiated cells

**Fig. 2:**
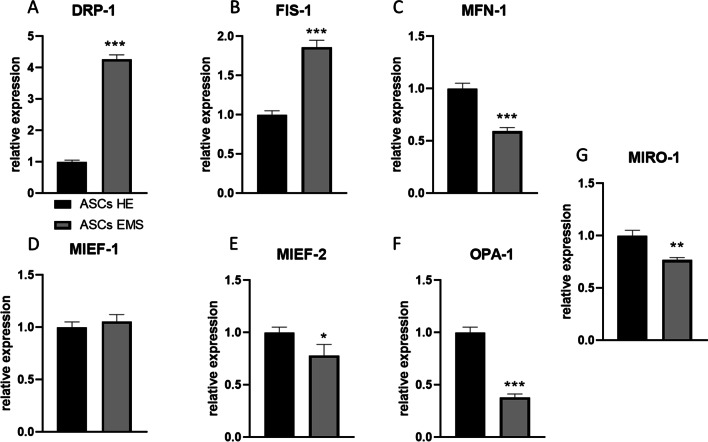
Expression of mitochondrial dynamics genes. Expression pattern of DRP-1 (**a**), FIS-1 (**b**), MFN-1 (**c**), MIEF-1 (**d**), MIEF-2 (**e**), OPA-1 (**f**) and MIRO-1 (**g**) was established by RT-qPCR. Results are expressed as mean ± SD; n = 3. ∗*p* < 0.05, ∗∗*p* < 0.01, and ∗∗∗*p* < 0.001

**Fig. 3:**
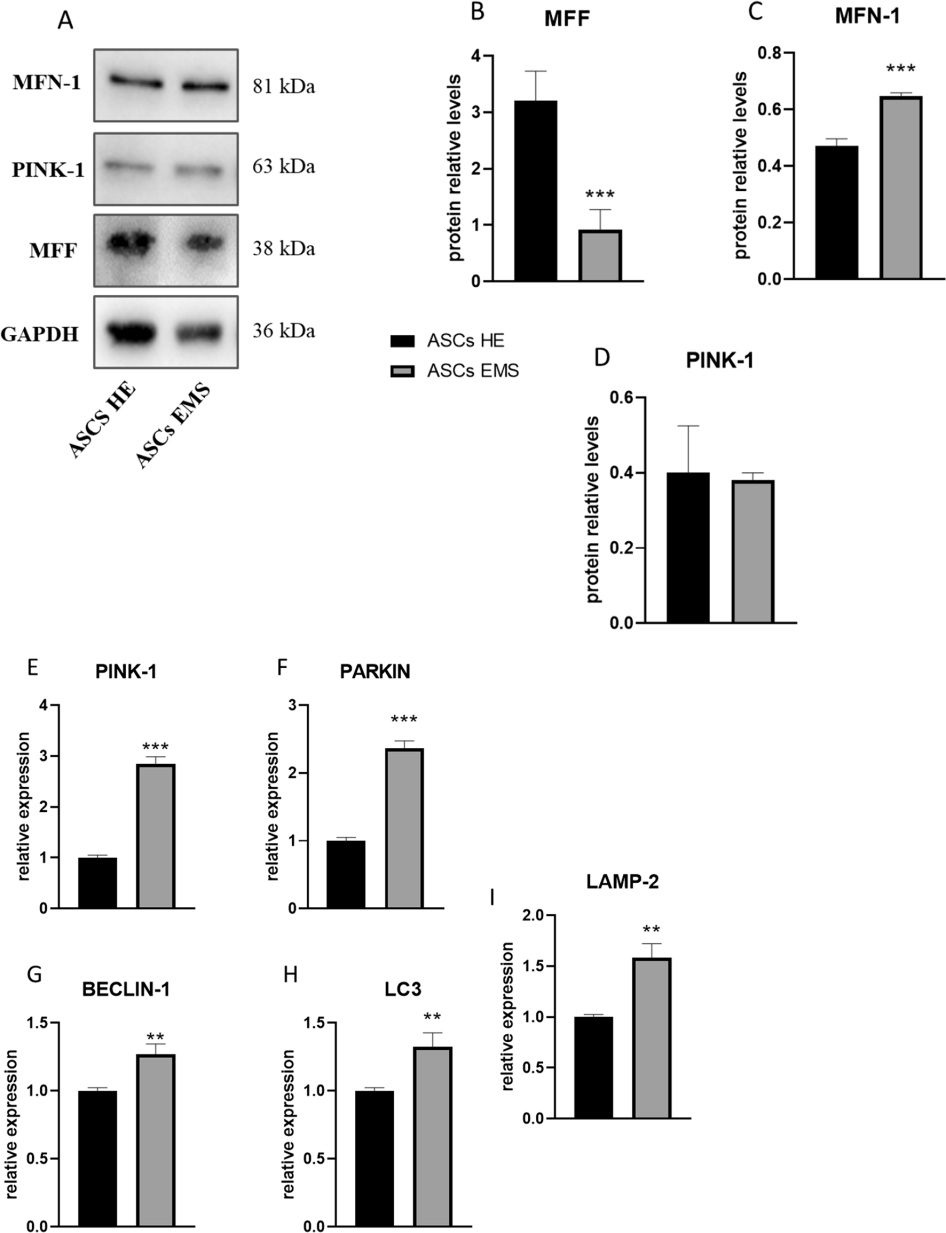
Evaluation of mitophagy and autophagy. Representative bands (**a**) show the results of western blotting for mitophagy related proteins. The data was quantified and the levels of MFF (**b**), MFN-1 (**c**), and PINK-1 (**d**) were established. Using RT-qPCR, the expression pattern of PINK-1 (**e**), PARKIN (**f**), BECLIN-1 (**g**), LC3 (**h**), and LAMP-2 (**i**) was evaluated. Results are expressed as mean ± SD; *n* = 3. ∗*p* < 0.05, ∗∗*p* < 0.01, and ∗∗∗*p* < 0.001

Expression of DRP-1 (Fig. 2a) and FIS-1 (Fig. 2b) was significantly upregulated (*p* < 0.001) in ASCs EMS. On the other hand, MFN-1 (Fig. 2c) mRNA levels were diminished in ASCs EMS (*p* < 0.001), while no differences in the expression of MIEF-1 (Fig. 2d) were noted. MIEF-2 (Fig. 2e, *p* < 0.05), OPA-1 (Fig. 1f, *p* < 0.001), and MIRO-1 (Fig. 1g, *p* < 0.01) expression was decreased in ASCs EMS. Furthermore, we performed the western blot analysis of proteins related to mitochondrial dynamics and representative bands are shown in Fig. 3a. Data was further quantified and the results revealed decreased levels of MFF (Fig. 3b, *p* < 0.001) and increased levels of MFN-1 (Fig. 3c, *p* < 0.001) in ASCs EMS. No differences were noted in the amount of PINK-1 (Fig. 3d) between the investigated groups. Furthermore, the expression of mito and autophagy related genes was investigated with RT-qPCR. We have found significantly increased expression of PINK-1 (Fig. 3e, *p* < 0.001), PARKIN (Fig. 3f, *p* < 0.001), BECLIN-1 (Fig. 3g, *p* < 0.01), LC3 (Fig. 3h, *p* < 0.01) and LAMP-2 (Fig. 3i, *p* < 0.01) in ASCs EMS in comparison to ASCs HE.

### Evaluation of adipogenesis effectiveness

**Fig. 4:**
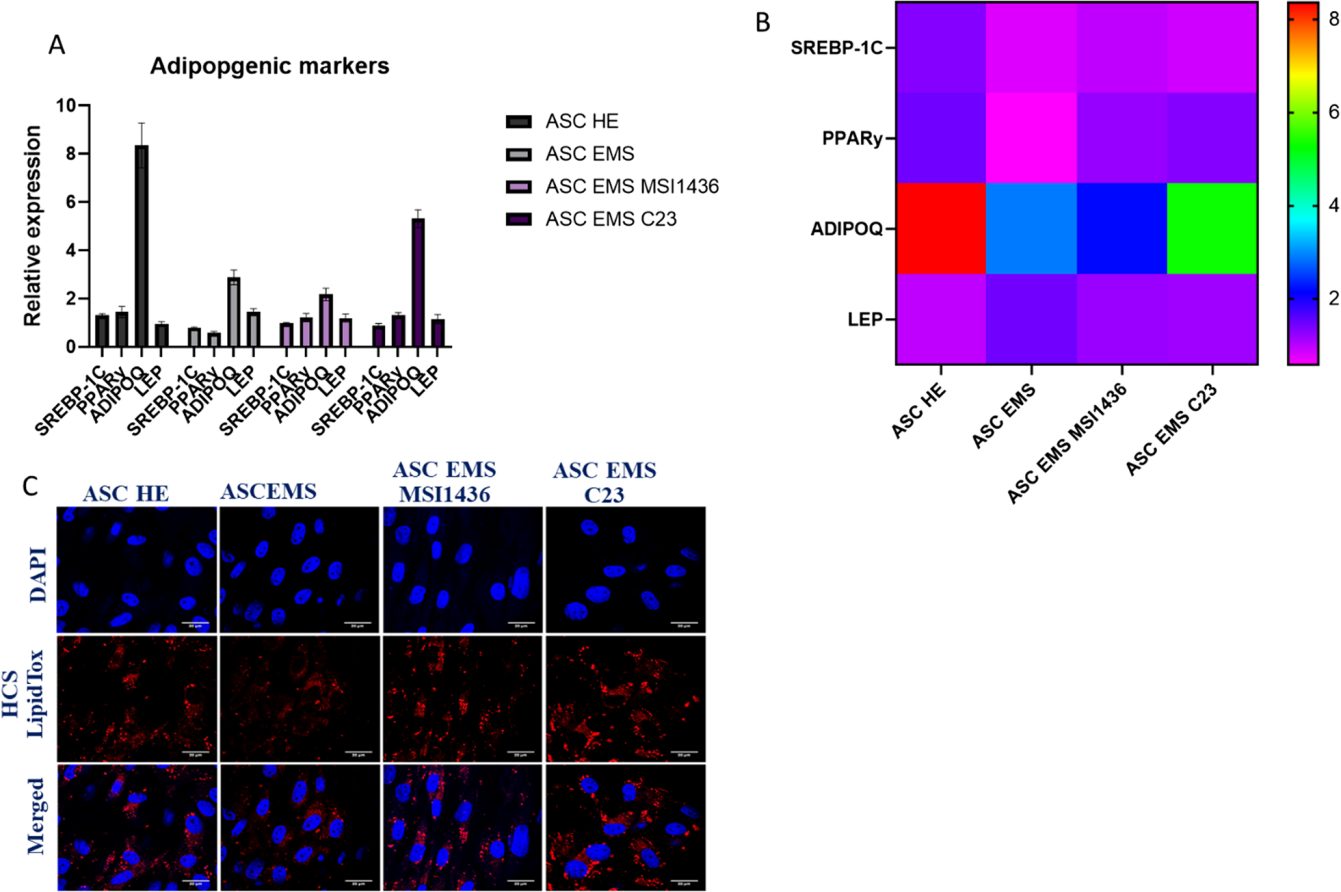
Evaluation of adipogenesis efficiency. Expression of key adipogenic genes was established with RT-qPCR (**a**). Obtained data were also presented in the form of heat map (**b**). Accumulation of lipid droplets within the cells was confirmed with LipidTox staining (**c**). Results are expressed as mean ± SD; *n* = 3

In the next steps of the experiments, we investigated the adipogenic differentiation of ASCs in the context of mitochondrial dynamics in standard culture conditions and during PTP1B and LMPTP inhibition. PTP1B activity was diminished by using MSI1436 inhibitor, while the activity of LMPTP was reduced with C23 inhibitor. Cells were cultured in adipogenesis-stimulating conditions and then, we have tested using RT-qPCR the expression pattern of key adipogenesis regulatory genes in the investigated cells. Results were displayed as box plot (Fig. 4a) and heatmap (Fig. 4b). Among all investigated groups, adiponectin was expressed as the highest rate. Interestingly, in ASCs adiponectin levels were reduced in comparison to ASCs HE, however, application of C23 in EMS cells significantly increased its expression. Furthermore, we have stained cells with LipidTox (Fig. 4c) to visualize the formation of lipid droplets within the cells.

### Evaluation of mitochondria condition and antioxidative defence during adipogenesis

**Fig. 5:**
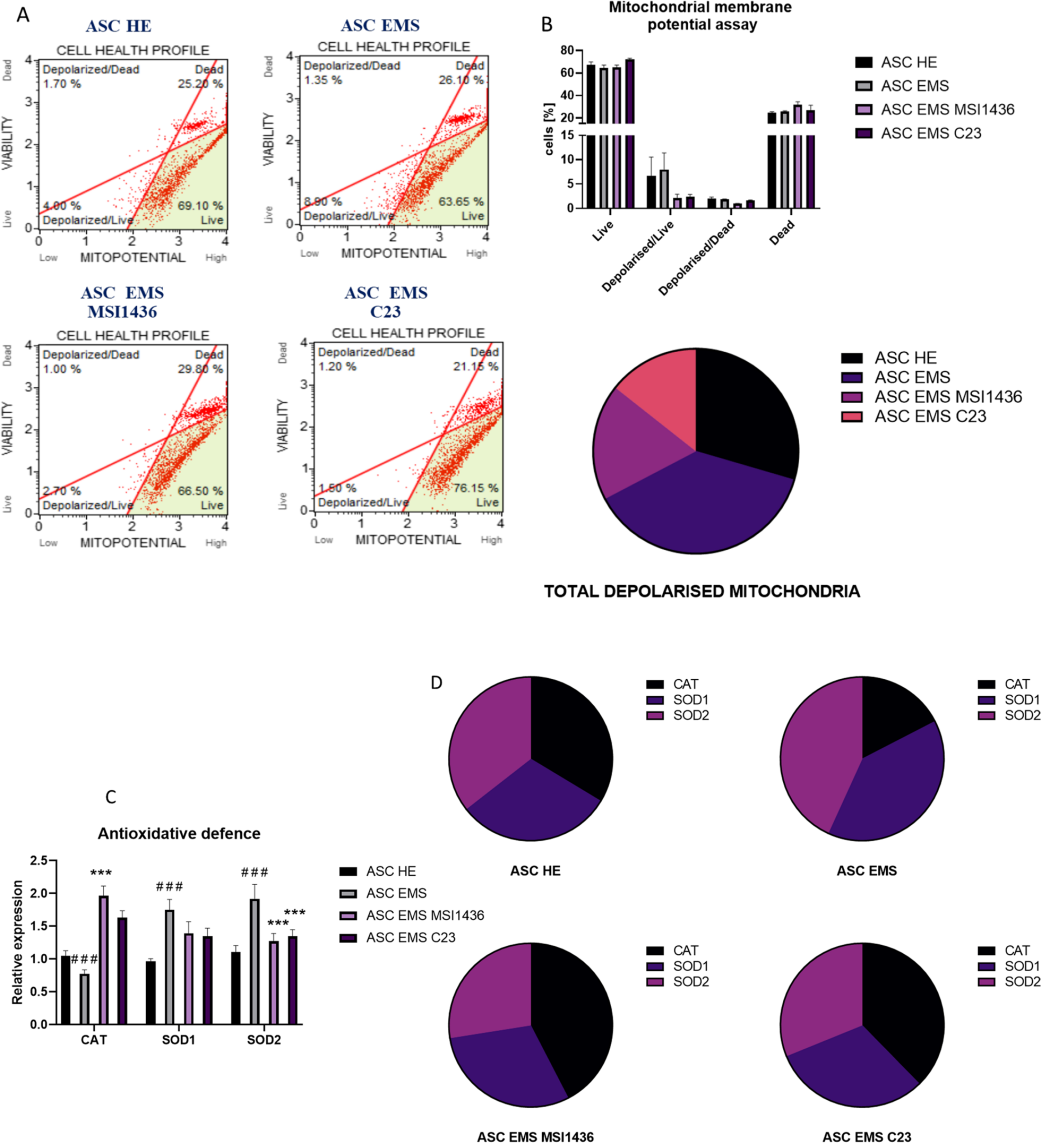
Evaluation of mitochondria condition and antioxidative defence during adipogenesis. Mitochondrial membrane potential was evaluated with MUSE cell analyser (**a**) allowing for the determination of the number of cells with depolarised mitochondria (**b**). Antioxidative properties of the cells were tested by the determination of CAT, SOD1, and SOD2 expression (**c**). Additionally, the obtained results were shown in part of a whole graph (**d**). Results are expressed as mean ± SD; *n* = 3. Statistical significance indicated as asterisk (*) when comparing the result to ASCs HE and as number sign (#) when compared to ASCs EMS. ∗∗∗*p* < 0.001

Mitochondrial membrane potential was established with MUSE cell analyser and representative dot plots are shown in Fig. 5a. Furthermore, the data was quantified and displayed as a part of the whole graph (Fig. 5b). ASCs EMS were characterised by an increased number of depolarised organelles in comparison to ASCs HE. Interestingly, application of both MSI1436 and C23 decreased the number of damaged mitochondria in ASCs EMS cells. Furthermore, to test the antioxidative properties of the investigated cells, we have analysed with RT-qPCR the expression of CAT, SOD1, and SOD2 (Fig. 5c). The highest expression of CAT was observed in ASCs EMS treated with MSI1436, while SOD1 and SOD2 in ASCs EMS. Additionally, the obtained data was also displayed as a part of a whole graph (Fig. 5d).

### Analysis of mitochondrial dynamics during adipogenic differentiation

**Fig. 6:**
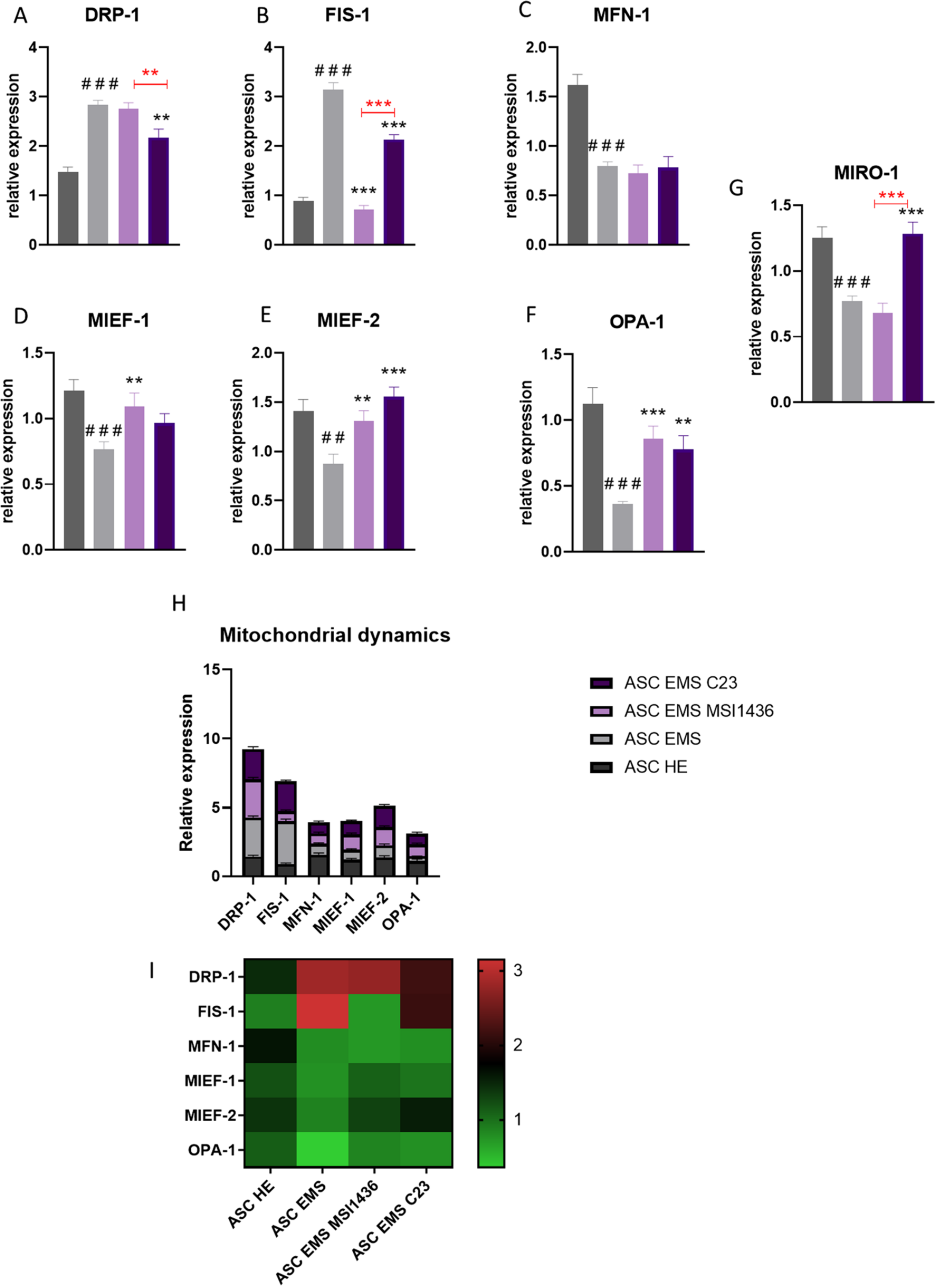
Analysis of mitochondrial dynamics during adipogenic differentiation. Expression of genes involved in the modulation of mitochondrial dynamics-DRP-1 (**a**), FIS-1 (**b**), MFN-1 (**c**), MIEF-1 (**d**), MIEF-2 (**e**), OPA-1 (**f**) and MIRO-1 (**g**) was established by RT-qPCR. Additionally, the obtained data were displayed as a grouped summary data (**h**) and heat map (**i**). Results are expressed as mean ± SD; *n* = 3. ∗*p* < 0.05, ∗∗*p* < 0.01, and ∗∗∗*p* < 0.001. Statistical significance indicated as asterisk (*) when comparing the result to ASCs HE and as number sign (#) when comparing to ASCs EMS

Expression of genes related to the regulation of mitochondrial dynamics was established with RT-qPCR. DRP-1 (Fig. 6a) expression was increased in ASCs EMS (*p* < 0.001), but application of C23 inhibited its expression (*p* < 0.01). Expression of FIS-1 (Fig. 6b) was upregulated in ASCs EMS but application of inhibitors significantly reduced its expression. On the other hand, the inhibitors did not influence the expression of MFN-1 (Fig. 6c). mRNA levels of MIEF-1 (Fig. 6d) were downregulated in ASCs EMS but application of MSI1436 augments expression. ASCs and EMS were characterised by decreased expression of MIEF-2 (Fig. 6e) and OPA-1 (Fig. 6f), but application of both MSI1436 and C23 enhanced their expression. Expression of MIRO-1 (Fig. 6g) was enhanced in cells treated with C23. Obtained data as also displayed as grouped summary data (mean and SD) (Fig. 6h) and heat map (Fig. 6i).

### Analysis of mitochondrial morphology, mitophagy, and autophagy during adipogenic differentiation

**Fig. 7:**
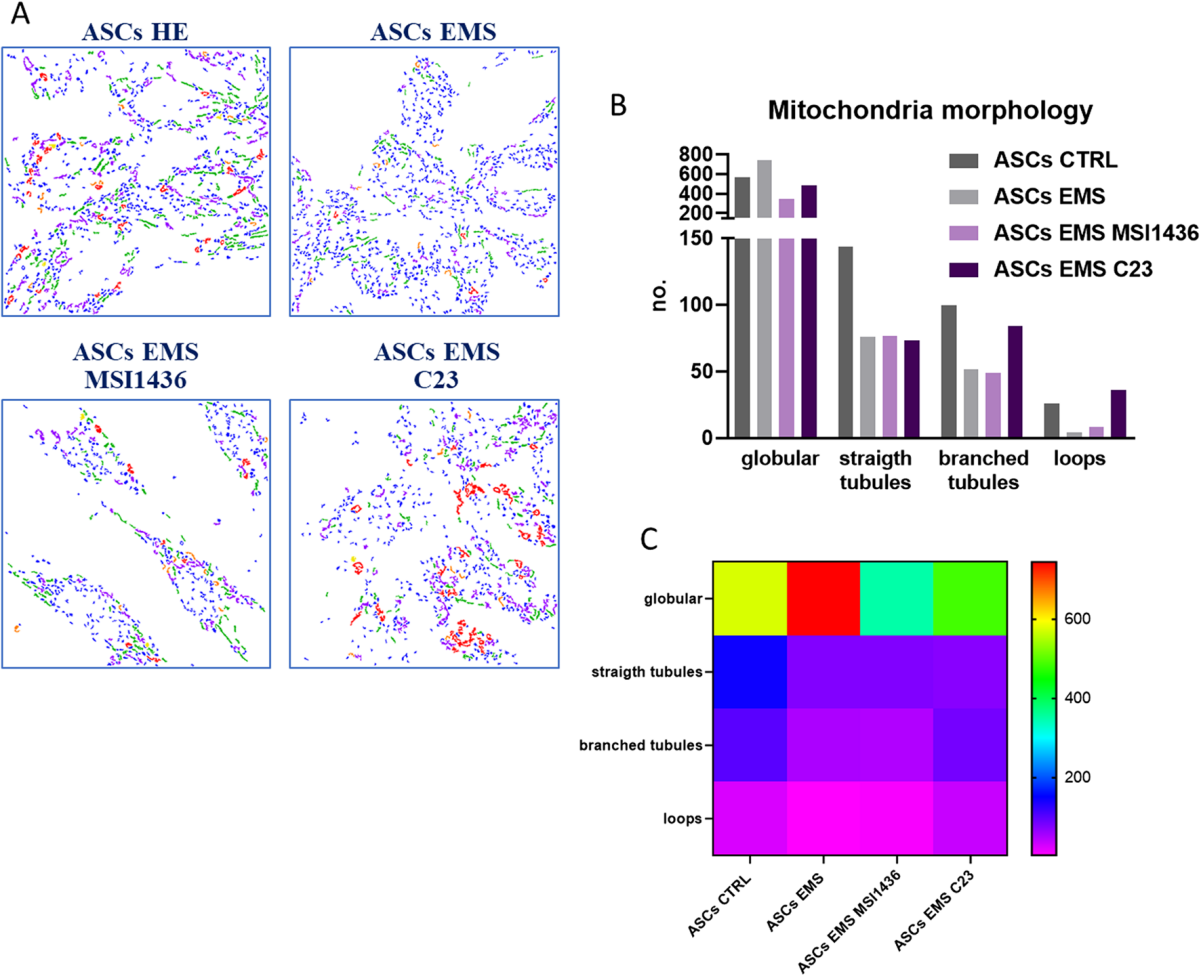

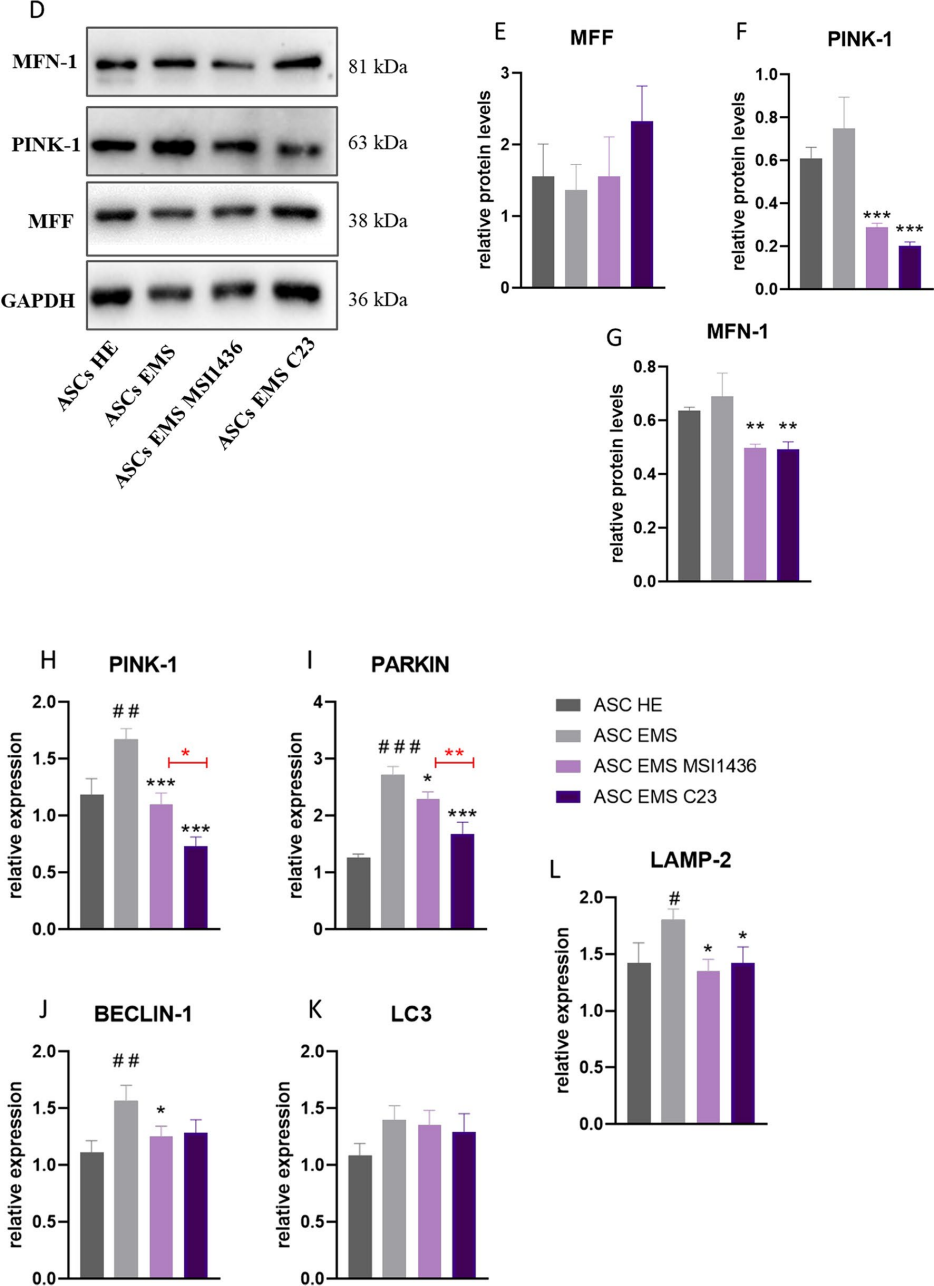
Analysis of mitochondrial morphology, mitophagy, and autophagy during adipogenic differentiation. Images showing mitochondrial networks by cells analysed by MicroP software (**a**). Based on the MicroP data, mitochondrial morphology in cells was established (**b**). Additionally, the data was displayed as a heat map (**c**). Representative bands (**d**) show the results of western blot. The amount of MFF (**e**), PINK-1 (**f**), and MFN-1 (**g**) was established. Using RT-qPCR, expression of genes involved in mitophagy and autophagy-PINK-1 (**h**), PARKIN (**i**), BECLIN-1 (**j**), LC3 (**k**) and LAMP-2 (**l**) was investigated. Results are expressed as mean ± SD; *n* = 3. ∗*p* < 0.05, ∗∗*p* < 0.01, and ∗∗∗*p* < 0.001. Statistical significance indicated as asterisk (*) when comparing the result to ASCs HE and as number sign (#) when compared to ASCs EMS

Morphology of mitochondria in cells which underwent adipogenic differentiation and LMPTP or PTP1B inhibition was investigated with MicroP software. Representative pictures are shown in Fig. 7a. Quantitative data (Fig. 7b) revealed that ASCs EMS were characterised by an increased number of globular, fragmented organelles, while the presence of long, connected mitochondria were characterised for ASCs HE. Interestingly, application of C23 increased the formation of branched, connected mitochondrial nets in cells. Furthermore, the obtained data was also presented as a heat map (Fig. 7c). Additionally, using western blot, we established the amount of proteins related to mitochondrial dynamics in the investigated cells. Representative bands are shown in Fig. 7d. No statistically significant differences were noted in the levels of MMF (Fig. 7e), while the amount of PINK-1 (Fig. 7f) was significantly (*p* < 0.001) reduced in cells treated with MSI1436 and C23 in comparison to their untreated counterparts. Similar phenomenon was observed for MFN-1 protein (Fig. 7g).

Additionally, using RT-qPCR, we have investigated the expression of genes involved in mito- and autophagy. We have found that application of MSI1436 and C23 significantly reduced expression of PINK-1 (Fig. 7h), PARKIN (Fig. 7i), and BECLIN-1 (Fig. 7j). No differences in the expression of LC3 (Fig. 7k) were observed between the investigated groups. Application of MSI143 and C23 also decreased the expression of LAMP-2 (Fig. 7l).

### Analysis of mitochondria metabolism during adipogenic differentiation

**Fig. 8:**
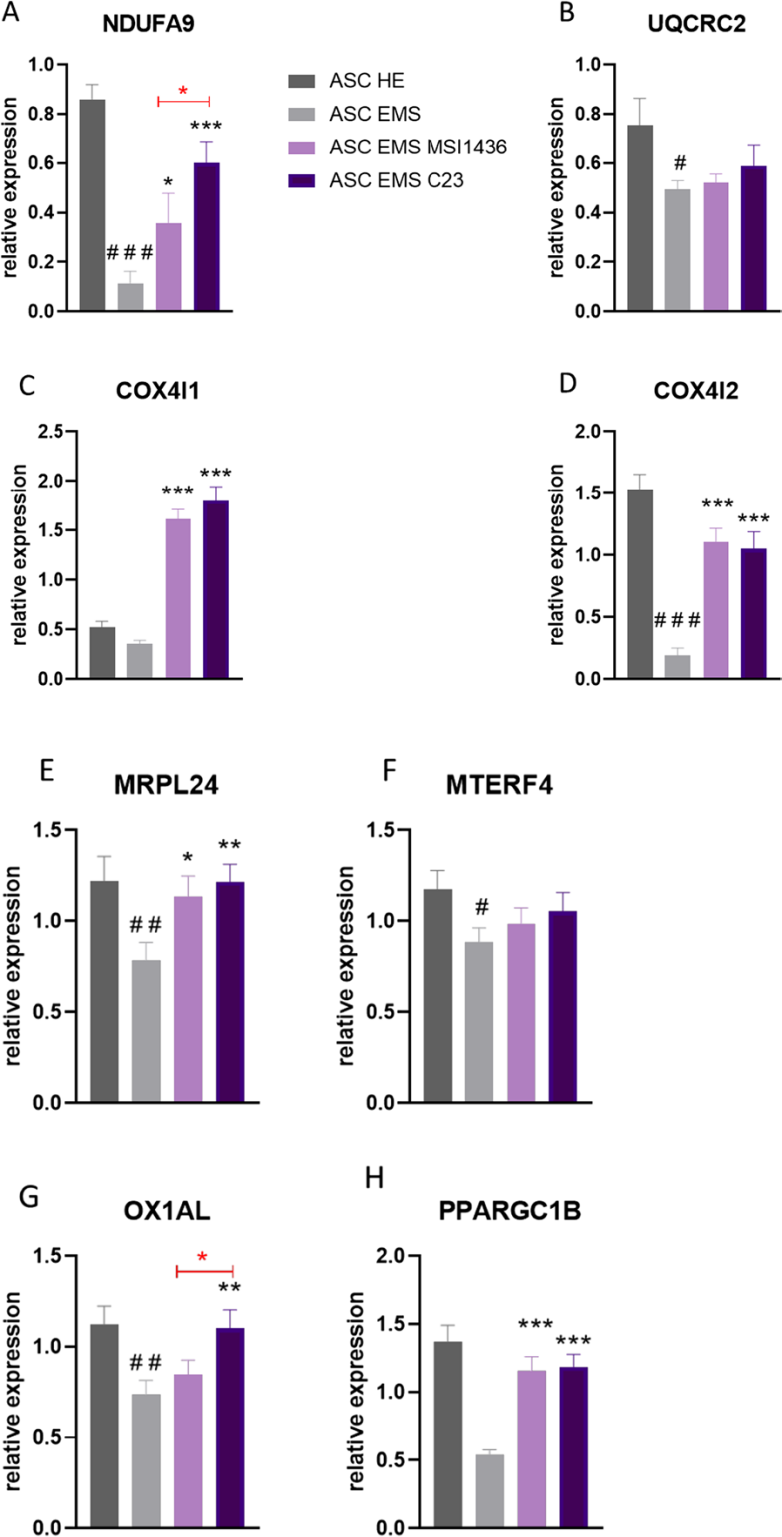

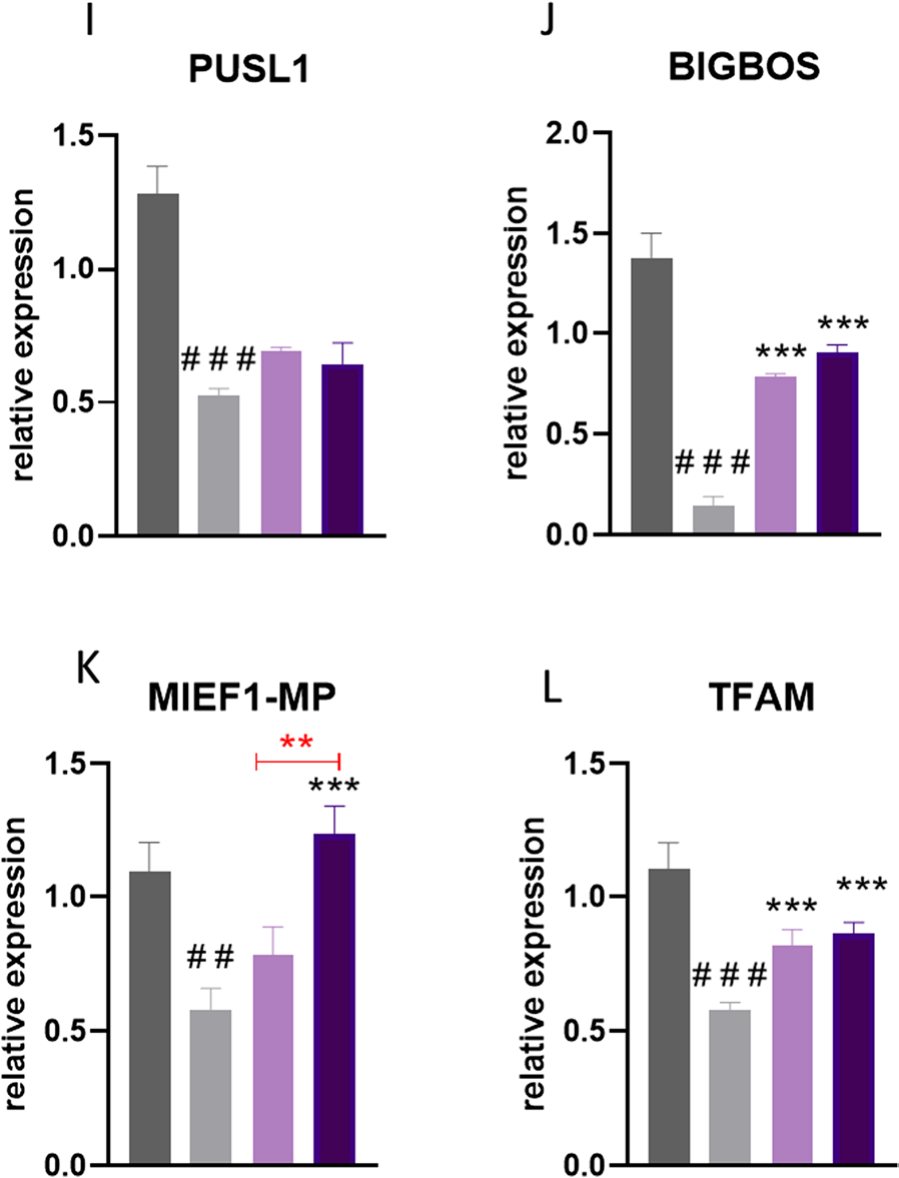
Analysis of mitochondria metabolism during adipogenic differentiation. Using RT-qPCR, we analysed expression of genes related to mitoribosomes and mitochondrial oxidative phosphorylation (OXPHOS) system-NDUFA9 (**a**), UQCRC2 (**b**), COX4I1 (**c**), COX4I2 (**d**), MRPL9 (**e**), MTERF4 (**f**), OX1AL (**g**), PPARGC1B (**h**), PUSL1 (**i**), BIGBOS (**j**), MIEF1-MP (**k**) and TFAM (**l**) in cells cultured in adipogenic conditions. Results are expressed as mean ± SD; *n* = 3. ∗*p* < 0.05, ∗∗*p* < 0.01, and ∗∗∗*p* < 0.001. Statistical significance indicated as asterisk (*) when comparing the result to ASCs HE and as number sign (#) when compared to ASCs EMS

Using RT-qPCR, we analysed the expression of genes related to mitoribosomes and the mitochondrial oxidative phosphorylation (OXPHOS) system in cells cultured in adipogenic conditions. We have found that both MSI1436 and C23 significantly enhanced the expression of NDUFA9 (Fig. 8a). UQCRC2 (Fig. 8b) mRNA levels were diminished in ASCs EMS when compared to ASCs HE and inhibitors did not influence their expression. Application of MSI1436 and C23 significantly increased expression of COX4I1 (Fig. 8c) and COX4I2 (Fig. 8d) in ASCs EMS (*p* < 0.001). Inhibitors also increased the expression of MRPL23 (Fig. 8e) while did not influence the expression of MTERF4 (Fig. 8f). C23 application resulted in enhanced expression of OX1AL (Fig. 8g, *p* < 0.01). Both inhibitors upregulated the expression of PPARGC1B (Fig. 8h) in ASCs EMS (*p* < 0.001) while did not change the expression of PUSL1 (Fig. 8i). BIGBOS (Fig. 8j) expression was enhanced by the application of both inhibitors in ASCs EMS, while C23 also enhanced the expression of MIEF1-MP (Fig. 8k). We also observed that MSI1436 and C23 enhanced the expression of TFAM (Fig. 8l) in ASCs EMS.

## Discussion

Inhibition of protein tyrosine phosphatase 1B (PTP1B) improves insulin sensitivity and therefore might protect against the development of obesity, insulin resistance, and insulin dysregulation—the common features of metabolic syndrome or type 2 diabetes [29, 39]. The two processes, hypertrophy and hyperplasia, are directly involved in adipose tissue metabolic deterioration during metabolic disorders. Our and others studies have demonstrated that adipose tissue collected from MetS or EMS patients is abundantly infiltrated by macrophages and mononuclear cells with a reduced number of regulatory lymphocytes (Tregs), which protect against inflammation development [2, 40]. Highly inflamed adipose tissue secretes a number of proinflammatory cytokines which negatively affect not only adipocytes but also residing within adipose stem progenitor cells (ASCs) [19]. Our previous studies have shown that ASCs, which gives rise to mature adipocytes, due to the defective adipogenic differentiation process, plays a fundamental role in adipose tissue hyperplasia and hypertrophy [2, 41]. In this study, we showed that MSI1436 and C23 improved adipogenic differentiation of ASCs isolated from EMS (ASC EMS) horses by the modulation of mitochondrial biogenesis and dynamics as well as reducing the level of oxidative stress. We observed that both inhibitors enhance the activity of CCAAT/enhancer-binding protein C/EBPα and peroxisome proliferator-activated receptor γ (PPARγ), two master transcription factors for terminal adipocyte differentiation. At the same time, we observed that MSI1436 and C23 reduce the expression of both AKT1 as well as AKT2, which mediates adipogenesis in adipocytes. Our and others early studies showed that under MetS or EMS condition, the expression of C/EBPα and PPARγ are drastically reduced, which causes lipodystrophy and adipose tissue inflammation as well as promotes the accumulation of ROS and NO—two inducers of defective adipogenic differentiation [42]. Recently, Dubois [43] has shown that reduced expression of transcripts i.e., C/EBPα, and PPARγ results in the development of hypertrophic and insulin-resistant adipocytes. This stands in good agreement with our recent observations, since we have demonstrated that ASC EMS under adipogenic differentiation gives rise to an enlarged the population of adipocytes. The correlation between the expression of C/EBPα and PPARγ adipocytes morphotype as well as adiponectin and leptin concentrations has been shown in EMS patients [19]. Interestingly, we have found that C23, in contrast to MSI1436, significantly improves the expression of adiponectin in ASC EMS under differentiation conditions. At the same time, we observed that both inhibitors of PTP1B significantly reduced the activity of leptin. The observed phenomenon shed a promising light for the application of both inhibitors as protective agent against insulin resistance development, since the advantage of adiponectin over leptin has been shown to protect against metabolic disorders development [44]. Moreover, the adiponectin-leptin axis has been shown to be strongly associated with dysfunction of adipose tissue, and a low ration of ALA may contribute to the development of oxidative stress and inflammation—to critical hallmarks of the EMS [45]. Here we have found that two isoforms of superoxide dismutase (SOD), i.e., SOD1 and SOD2, when exposed to MSI1436 as well as C23 are stabilized at the level characteristic for healthy ASCs. In our earlier study, we demonstrated that ASC EMS are losing their multipotent character by excessive accumulation of ROS which induces mitochondrial stress [22]. These in turn lead to impaired mitochondrial biogenesis and dynamics and impaired mitophagy, which prevents "regeneration" of damaged mitochondria. Moreover, we observed that together with SOD, catalase activity was highly upregulated, which might serve as an antioxidant protection against insulin resistance development. Recently, Amos and Colleges [46] showed that in “stress-less” mouse model (“Bob-Cat”), which has decreased oxidative stress, catalase mRNA expression was increased in key metabolic tissues including adipose, liver, intestinal mucosa, and brain.

Mitochondria have been shown to play a critical role in progenitor stem cell differentiation potential, multipotency as well as sensitivity to insulin [25, 47]. Mitochondrial dynamics is modulated during differentiation of stem cells, allowing for the alternation of the bioenergetic profile. It was shown by Forni et al. [48] found that mitochondrial fusion is crucial to adipogenic differentiation of murine stem cells. It stands in a good agreement with our data as we observed an increased number of fragmented mitochondria in ASC EMS, however, treatment of cells with MSI1436 and C23 enhanced the formation of connected, branched organelles and the expression of fusion genes-MFN-1 and MFN-2. Mitochondria play a fundamental role in cellular bioenergetics, producing the majority of adenosine triphosphate molecules by the oxidative phosphorylation system (OXPHOS). In particular, OXPHOS so far has been shown to be strongly involved in the regulation of neuronal development, plasticity, and differentiation [49]. In this study, for the first time, we showed that ASC EMS under adipogenic differentiation conditions exhibits reduced expression of NDUFA9, UQCRC2, and COX4I1 and COX4I2-the master regulators of OXPHOS. We have found that MSI1436 as well as C23 activates OXPHOS in ASC EMS contributing to enhanced adipogenesis. Recently, Ryu and Colleges have showed that mouse derived ASCs with lower levels of mtDNA-encoded OXPHOS subunits are characterized by an impaired adipogenesis process and give rise to impaired adipocytes which finally lead to the development of defective adipose tissue. Recent data suggests that metabolic syndrome is strongly associated with changes in mitochondrial metabolism, which in turn might lead to obesity or insulin resistance development [50]. In this study, we have found that MSI1436 as well as C23 improves the expression of MRPL24, MTERF4, and OX1AL—the master regulators of mitochondrial ribosomal proteins (MRP). Kenmochi et al. [51] noted that chosen MRPs are associated with disorders including retinitis pigmentosa or Usher Syn-drome. Here, for the first time, we showed that the activity of MRPs are significantly reduced in ASC EMS, which suggests a critical role of mitochondrial ribosomal proteinS in the development of adipose tissue dysfunction and insulin resistance. Recently, it was proposed that the reduced expression of both mitochondrial transcription factors TFAM might be associated with different endocrine disorders [52]. In this study, we confirmed its reduced expression in ASC EMS. However, stimulation of ASC EMS with MSI1436 as well as C23 reversed that phenomenon. Moreover, ASC EMS under adipogenic differentiation when exposed to MSI1436 as well as C23 reduces the expression of PINK and PARKIN-the key regulators of mitochondrial degradation. The reduced activity of PINK-Parkin axis might suggest the protective role of MSI1436 and C23 against mitochondrial damage.

In this study, for the first time we demonstrated that ASC EMS due to exposure to oxidative stress under normal conditions are characterized by impaired cellular bioenergetics (OXPHOS), MRP as well as a highly activated PINK-Parkin axis which finally activates mitophagy to rescue damaged cells. However, the application of MSI1436 as well as C23 improves adipogenic differentiation of ASC EMS by activation of OXPHOS, mitochondrial biogenesis and dynamics, which finally improves insulin sensitivity. The presented result indicate that MSI1436 and C23 might protect against insulin resistance, obesity, and EMS development through the modulation of mitochondrial dynamics.

## Conclusion

 In this study, we have shown that inhibition of PTP1B and LMPTP with MSI1436 and C23, respectively, enhanced the adipogenic differentiation potential of ASC EMS through the modulation of mitochondrial dynamism. Obtained findings may contribute to the development of therapeutic strategies against insulin resistance aimed at the modulation of progenitor stem cell plasticity and as a consequence adipose tissue metabolism.

## Data Availability

The data that support the findings of this study are available from the corresponding author, upon reasonable request.

## Abbreviations

ASC: Adipose progenitor stem cells
C/EBP: CCAAT/enhancer-binding protein
C23: (N,N-diethyl-4-(4-((3-(piperidin-1-yl)propyl)amino)quinolin-2-yl) benzamide)
CREBP: CAMP-responsive element-binding protein
DAPI: Diamidino-2-phenylindole
DMEM: Dulbecco’s modified Eagle’s medium
EMS: Equine metabolic syndrome
FBS: Fetal bovine serum
HFD: High fat diet
IMBX: 1-Methyl-3-isobutylxantine
IRP: Insulin receptor
IRSP: Insulin receptor substrate pathway
LMPTP: Low molecular weight protein tyrosine phosphatase
MetS: Human Syndrome X
MMP: Mitochondrial membrane potential
MRP: Mitochondrial ribosomal proteins
NO: Nitric oxide
OXPHOS: Oxidative phosphorylation
PPARγ: Peroxisome proliferator-activated receptor γ
PS: Penicillin and streptomycin
PTP1B: Protein tyrosine phosphatase 1B
PTPs: Protein tyrosine phosphatases
PVDF: Polyvinylidene difluoride
ROS: Reactive oxygen species
RT-PCR: Real-time reverse transcription polymerase chain reaction
SOD: Superoxide dismutase
TCA: Tricarboxylic acid
Tregs: Regulatory lymphocytes
