# Correction: Inhibition of protein tyrosine phosphatase improves mitochondrial bioenergetics and dynamics, reduces oxidative stress, and enhances adipogenic differentiation potential in metabolically impaired progenitor stem cells

**DOI:** 10.1186/s12964-023-01055-x

**Published:** 2023-01-20

**Authors:** Katarzyna Kornicka-Garbowska, Lynda Bourebaba, Michael Röcken, Krzysztof Marycz

**Affiliations:** 1 Department of Experimental Biology, Wroclaw University of Environmental and Life Sciences, Norwida 27B Street, A7 Building, 50-375 Wroclaw, Poland; 2 International Institute of Translational Medicine, Malin, Jesionowa 11, 55-114 Wisznia Mała, Poland; 3 Faculty of Veterinary Medicine, Equine Clinic-Equine Surgery, Justus-Liebig University, 35392 Giessen, Germany

**Correction: Cell Commun Signal (2021) 19:106** 10.1186/s12964-021-00772-5

Following publication of the original article [1], the authors reported an error in the funding section. The research grant “Inhibition of tyrosine phosphatase as a strategy to enhance insulin sensitivity through activation of chaperone mediated autophagy and amelioration of inflammation and cellular stress in the liver of equine metabolic syndrome (EMS) horses” (Grant No. 2018/29/B/NZ7/02662), financed by The National Science Centre in Poland is removed from the Acknowledgments/Financial disclosure.

The authors would like to apologise for any inconvenience caused.
